# Clinical Approach to Vascular Calcification in Patients With Non-dialysis Dependent Chronic Kidney Disease: Mineral-Bone Disorder-Related Aspects

**DOI:** 10.3389/fmed.2021.642718

**Published:** 2021-05-19

**Authors:** Jordi Bover, Armando Aguilar, Carolt Arana, Pablo Molina, María Jesús Lloret, Jackson Ochoa, Gerson Berná, Yessica G. Gutiérrez-Maza, Natacha Rodrigues, Luis D'Marco, José L. Górriz

**Affiliations:** ^1^Department of Nephrology, Fundació Puigvert, IIB Sant Pau, Universitat Autònoma, REDinREN, Barcelona, Spain; ^2^Department of Nephrology, Instituto Mexicano del Seguro Social, Hospital General de Zona No. 2, Tuxtla Gutiérrez, Mexico; ^3^Department of Nephrology, Hospital Universitario Dr Peset, Universidad de Valencia, REDinREN, Valencia, Spain; ^4^Division of Nephrology and Renal Transplantation, Department of Medicine, Centro Hospitalar Universitário Lisboa Norte, EPE, Lisboa, Portugal; ^5^Servicio de Nefrología, Hospital Clínico Universitario, INCLIVA, Universidad de Valencia, Valencia, Spain

**Keywords:** CKD, CKD-MBD, calcification, vascular calcification, phosphate, calciprotein particles

## Abstract

Chronic kidney disease (CKD) is associated with a very high morbimortality, mainly from cardiovascular origin, and CKD is currently considered in the high- or very high risk- cardiovascular risk category. CKD-mineral and bone disorders (CKD-MBDs), including vascular and/or valvular calcifications, are also associated with these poor outcomes. Vascular calcification (VC) is very prevalent (both intimal and medial), even in non-dialysis dependent patients, with a greater severity and more rapid progression. Simple X-ray based-scores such as Adragão's (AS) are useful prognostic tools and AS (even AS based on hand-X-ray only) may be superior to the classic Kauppila's score when evaluating non-dialysis CKD patients. Thus, in this mini-review, we briefly review CKD-MBD-related aspects of VC and its complex pathophysiology including the vast array of contributors and inhibitors. Furthermore, although VC is a surrogate marker and is not yet considered a treatment target, we consider that the presence of VC may be relevant in guiding therapeutic interventions, unless all patients are treated with the mindset of reducing the incidence or progression of VC with the currently available armamentarium. Avoiding phosphate loading, restricting calcium-based phosphate binders and high doses of vitamin D, and avoiding normalizing (within the normal limits for the assay) parathyroid hormone levels seem logical approaches. The availability of new drugs and future studies, including patients in early stages of CKD, may lead to significant improvements not only in patient risk stratification but also in attenuating the accelerated progression of VC in CKD.

## Introduction: Chronic Kidney Disease And Cardiovascular Disease

Chronic kidney disease (CKD) has become a major public health problem because of the associated high mortality and elevated cost of treatment for dialysis patients. Worldwide, it is estimated that about 500 million adults suffer from CKD (1), but prevalence varies widely between countries (2). In Spain, CKD prevalence is 15.1% (3), is more common in men, in patients with cardiovascular disease (CVD) (39.8%), and increases with age (37.3% in those ≥65 years) (3). Given this significant link between CKD and CVD, CKD is currently considered an independent cardiovascular risk factor (4). A similar scenario is described for diabetes (5), a leading cause of CKD. Moreover, an independent, graded association was observed between a reduced estimated glomerular filtration rate (eGFR) and the risk of death, CVD, and hospitalization in a huge community-based population (6). Consequently, the European Society of Cardiology and European Atherosclerosis Society (ESC/EAS) recently considered moderate (eGFR 30–59 ml/min/1.73m^2^) and severe (eGFR <30 ml/min/1.73m^2^) CKD to be in the high*-* and the very high risk category of cardiovascular risk factors (7).

Since the seminal report by Foley et al. (8), who noted an extremely high cardiovascular mortality even in young dialysis patients, the existence of non-traditional cardiovascular risk factors, including mineral metabolism parameters, has caught the attention of many. Subclinical atherosclerosis is very frequent in CKD patients, and its evolution is also closely linked with CKD progression and related to several CKD-associated factors (9, 10). Thus, bone was recently described as a new endocrine organ “at the heart” of CKD and mineral-bone disorders (CKD-MBD) (11). Subsequently, the presence (12, 13) and progression (14, 15) of vascular and valvular calcification (16), which are also associated with “normal” aging (17), were clearly linked to prognosis (18–22), including in non-dialysis CKD (ND-CKD) patients, especially if the coronary artery calcification (CAC) score was evaluated (20–24). Therefore, CKD could be viewed conceptually as an accelerator of traditional cardiovascular risk factors and vascular calcification (VC) (17). Thus, in this review we will focus on the CKD-MBD aspects of VC including potentially helpful treatment considerations.

## Chronic Kidney Disease-Mineral and Bone Disorders (Ckd-Mbd) and Vascular Calcification

The term CKD-MBD was first coined in 2006 (25). It described a systemic disorder due to CKD, manifested by mineral and bone abnormalities and/or extraskeletal calcifications (25). Several studies have demonstrated the high prevalence of cardiovascular calcifications in CKD, even in ND-CKD patients (20, 21, 26–30), with a greater severity (20, 31) and more rapid progression (32, 33). Furthermore, it has been suggested that these cardiovascular calcifications may be not only a useful prognostic tool but also relevant in guiding therapeutic interventions (19, 34, 35), as it will be reviewed in section Treatment Implications. However, we will first let readers understand the different diagnostic methods and the important contribution of CKD-MBD-related aspects in the pathophysiology of VC in CKD patients (section Pathophysiology of CKD-MBD-related Vascular Calcification).

In clinical studies, VC is frequently detected by multisliced computed tomography (CT) and less often by electron-beam CT (20, 36–38), multiterritorial vascular ultrasound (9), intravascular ultrasound (IVUS) or Virtual Histology® IVUS (39, 40), arteriography, or even positron emission tomography (PET) scans (41, 42). Thus, the prevalence of CAC has been recently systematically reviewed and authors found a variable prevalence ranging from 28 to 93% in predialysis patients (59% pooled prevalence) (20). High heterogeneity was present, at least partially related to ethnic and population differences (such as age or CKD stages) (20). However, simple X-rays may also be used despite their much lower sensitivity and variable associations with CAC (43). In fact, guidelines suggest that a lateral abdominal radiograph can be used to detect VC and an echocardiogram can be used to detect valvular calcifications, as reasonable alternatives to CT-based imaging (35, 44). These guidelines also suggest that patients with CKD G3a–G5D with known vascular or valvular calcification be considered at highest cardiovascular risk (evidence 2A) and that it is reasonable to use this information to guide CKD-MBD management (35, 44).

**Figure 1:**
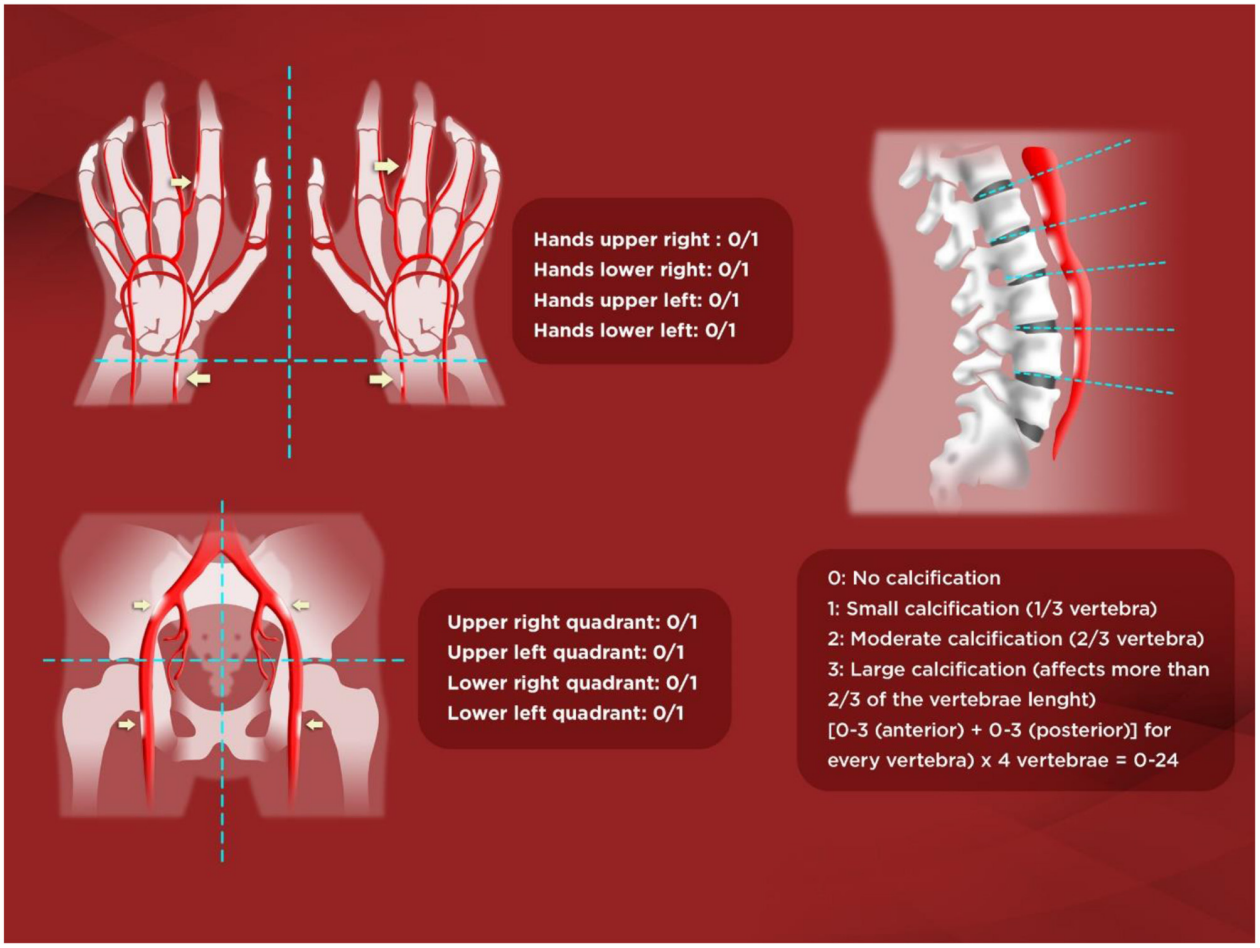
Adragão and Kauppila scores. ***Kauppila score (KS)***. The score is assigned from 1 to 3 [1: small calcification (1/3 of the vertebral length), 2: moderate (2/3), 3: large (> 2/3)] depending on the length of each plaque detected. The anterior and posterior part of the aorta shall be taken into consideration, associating them with the place where they are located, in front of the L1, L2, L3, or L4 vertebrae. With KS, a final score is achieved between *0 and 24 points*. ***Adrag*ã*o score (AS)***. The score is determined by the sum of the absence of calcification (0 points), or the presence of unilateral (1 point) or bilateral (2 points) linear calcification in each section. AS analyzes the calcification of iliac, femoral, radial, and digital arteries. The final value ranges from 0 to 8 points (0–4 in the pelvis and 0–4 in the hands).

Lateral abdominal X-ray is helpful not only to semiquantify an aortic risk score such as Kauppila's (KS) (45) (Figure 1) but also to detect unnoticed vertebral fractures, with potential therapeutic renewed interest in CKD-associated osteoporosis guidelines (35, 44, 46, 47). Spanish CKD-MBD guidelines also suggest use of the simpler Adragão's score (AS) (19, 48) (Figure 1). In their original study in hemodialysis patients, an AS≥3 was independently associated with coronary, peripheral, and vascular disease (48). Importantly, patients with an AS≥3 had a 2–4-fold higher risk of cardiovascular mortality, hospitalizations, and fatal or non-fatal cardiovascular events. In another study, it was found that an AS>3 was also significantly associated with pulse wave velocity (PWV), pulse pressure, and lower adjusted cumulative survival[hazard ratio (HR) = 3.31)] (19).

In the observational multicentre OSERCE-2 study (26), we prospectively analyzed VC in a cohort of 742 ND-CKD G3–G5 patients. VC was present in 79%, and VC was severe (AS≥3 or KS>6) in 47%. Age, phosphate (P) levels, and diabetes were independently associated with AS≥3. After a median follow-up of 35 months, AS≥3 but not KS>6 was independently associated with all-cause (HR = 2.07) and cardiovascular (HR = 3.46) mortality, and a shorter hospitalization event-free period (HR = 1.14). Moreover, only AS based on hand-X-ray only (AS-hands) (Figure 1) showed a significant correlation with parathyroid hormone (PTH) levels and renal function. Interestingly, the group AS≥3 showed more than double the risk of all-cause mortality, whereas the risk increased 5-fold using AS-hands. Moreover, AS≥3 but *not* KS>6 independently predicted all-cause, cardiovascular mortality and hospitalization, and AS-hands ≥1 was also an independent predictor of cardiovascular mortality and hospitalization-related outcomes. Nevertheless, other authors have described significant associations with hard-outcomes in ND-CKD patients evaluated for aortic, pelvic or even breast artery calcifications, and significant differences were also present among distinct vascular beds and associated factors (43, 49–52).

We therefore confirmed the potentially better predictive ability of simple X-rays with the AS (vs. KS), and extended its value into the ND-CKD population. Moreover, the even simpler AS-hands demonstrated an important predictive ability, probably because the radial and cubital arteries are muscular arteries with a greater tendency to calcification of the medial layer (arteriosclerosis), especially affected in CKD, as opposed to the elastic abdominal aorta, which may be more prone to intimal calcification (atheromatosis) (26, 48, 53).

Medial and intimal VC lead to different comorbidities: medial calcification (Mönckeberg's disease) induces arterial stiffness and consequently an increased PWV, left ventricular hypertrophy and heart failure, whereas intimal calcification is associated with ischemic episodes and is pathophysiologically characterized by endothelial damage, lipid accumulation, and infiltration of inflammatory cells. These data support the concept that VC (especially in CKD patients) is not just a single entity but the consequence of a wide range of different biological processes (54), affecting both intimal and medial layers, and it is difficult to study separately because common radiological techniques do not allow clear distinction between them (55, 56). Reliable differentiation between medial and intimal VC can only be achieved pathomorphologically (57). Moreover, VC is not exclusive to CKD and is especially prevalent in diabetes and during the aging process (also accelerated in CKD patients) (17).

Nevertheless, while the high prevalence and prognostic value of VC in CKD patients are now well-established, the clinical usefulness of early screening is still controversial since it is far from proven that early diagnosis permits beneficial actions that improve outcomes (e.g., regarding different P binders or vitamin D (VD) derivatives).

## Pathophysiology of CKD-MBD-Related Vascular Calcification

CKD-MBD-related factors are obviously not the only mechanisms responsible for VC but, as mentioned before, CKD acts as an accelerator of the VC process (17). Moreover, VC is not only a passive process of excess calcium (Ca) and P deposition (enhanced in CKD due to the loss of excretory function) but also an active process where transdifferentiation of vascular smooth muscle cells (VSMCs) from a contractile to a secretory calcific phenotype plays an important role (53, 54). This is probably magnified by CKD-MBD-related factors (11, 17). These VSMCs can also display adipogenic and macrophagic features (58–60). Moreover, adventitial Gli1+ mesenchymal stem-cells serve as progenitors of VSMC which migrate into the media and neointima during arteriosclerosis and atheromatosis (61).

**Figure 2:**
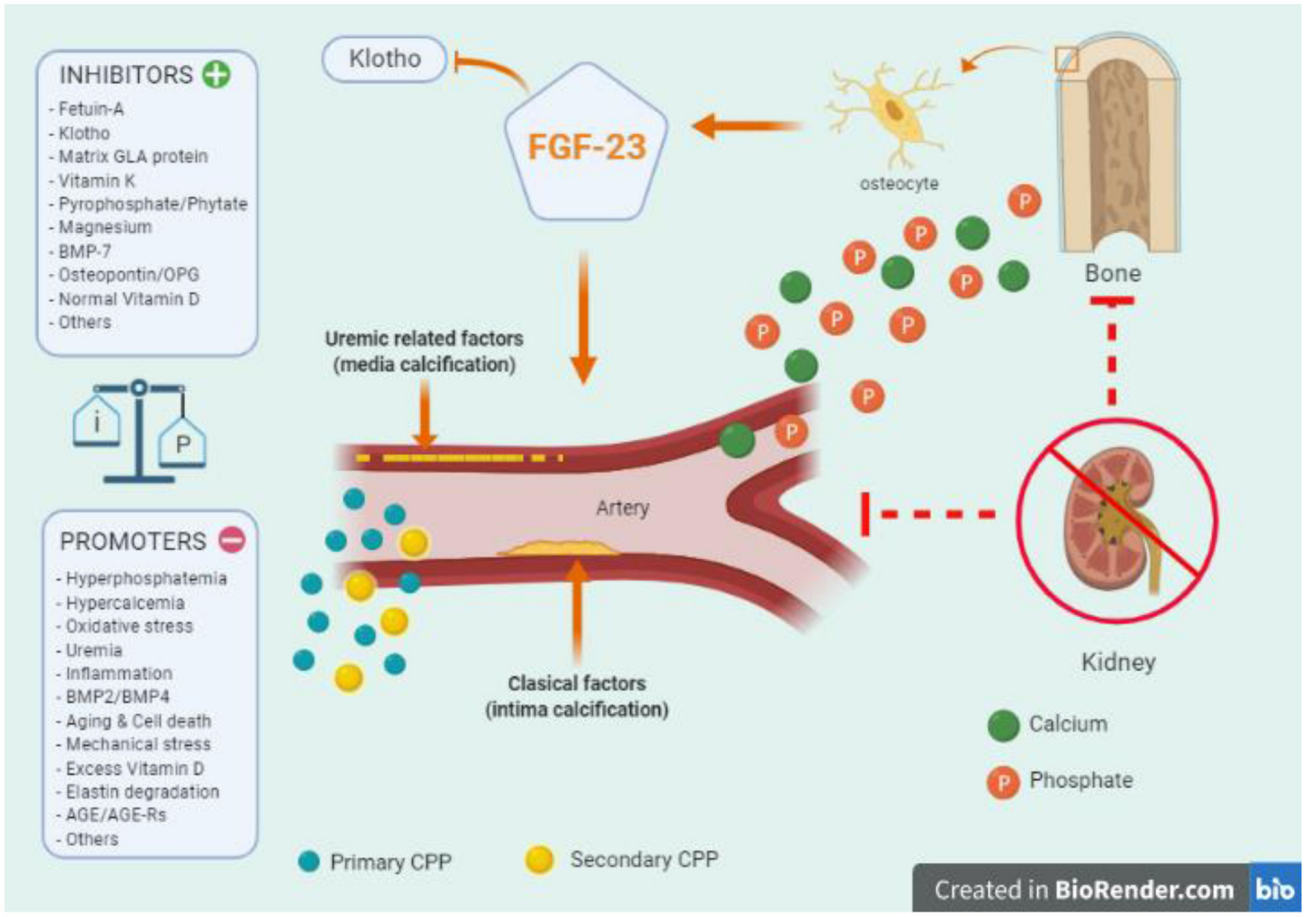
Key aspects of vascular calcification (VC) in chronic kidney disease (CKD): the important balance between calcification contributors and inhibitors (tissue or circulating factors) in the pathophysiology of vascular calcification. The altered balance between calcification contributors and inhibitors makes CKD patients more or less susceptible to accelerated calcifications. Of note, CKD patients exhibit both types of VC, intimal atheroma calcification, and medial arteriosclerosis. A key role in the initiation and propagation are calcium (green) and phosphate (orange) ions. The passive formation of calciprotein nanoparticles (CPP) act as active precursors of micro-calcification. Initially, the small calcium/phosphate complex can be removed by Fetuin-A, which can eventually become saturated leading to primary CPP (light blue) that can develop secondary CPP particles (yellow). Later, phosphate entries into vascular smooth muscle cells producing osteochondrocytic transdifferentiation (vascular “ossification”). Many of the contributors may also promote this phenotypic osteochondrocytic transdifferentiation. Among them, tissue non-specific alkaline phosphatase (TNAP), aldosterone, parathyroid hormone, activin-A, collagen I, osteocalcin, osteonectin, oxidized-low density lipoproteins, have been associated with vascular calcification. On the other hand, parathyroid hormone related-peptide, collagen IV, high density lipoproteins, nitric oxide, and others, have been described as potential inhibitors. At the bone level, the role of FGF-23 and the co-factor Klotho are relevant in all the process, and a genetic background (e.g., proteins derived from Ank, Npps, ENPP1 genes) should also be considered. BMP, Bone Morphogenetic Proteins; TNF-α, Tumor Necrosis Factor- α; IL, Interleukin; AGE, Advanced-Glication End-products; AGE-Rs, AGE-receptors.

Multiple physiopathological mechanisms and pathways are involved in the process of VC, but a comprehensive review is beyond the goals of this article, and we therefore refer interested readers to other references (62–67). Interestingly, some critical factors might play a double and sometimes opposite roles in terms of vascular health, such as the effects of high and low PTH levels on stem-cells (68, 69), or the roles of fibroblast growth factor-23 (FGF23) (70), VD (71), and calcimimetics (72). Importantly, in addition to a genetic background, the altered balance between calcification contributors (73–80) (Figure 2) and circulating or tissue calcification inhibitors (81–88) (Figure 2) makes CKD patients more or less susceptible to accelerated VC. Different comorbid pathologies associated with intimal atheroma calcification (ischemic events), arteriosclerosis (arterial stiffening), and heart valvular calcification may be overlapping, and CKD predisposes to all these types of VC (54, 64). Finally, the revolution in omics technology (genomics, epigenomics, transcriptomics, proteomics, and metabolomics) has resulted in the accumulation of cellular and molecular-level data (63, 67) which will provide novel approaches on the mechanisms associated with VC (63, 67). Although clinical application is still in its early stages, CKD may become an interesting model to analyse (17, 63), especially considering that as many as 17% of dialysis patients show no calcification (89).

Central to the increasing knowledge on mechanisms involved in VC is the discovery that calcifying extracellular vesicles act as active precursors of cardiovascular microcalcification in diverse vascular beds (90). Novel *in vitro* assays may quantify the propensity for VC in serum (as a composite measure capturing a global effect) by evaluating the semimaximal transformation time (T_50_) from the so-called primary to secondary calciprotein particles (CPP) when challenged with additional Ca and P (91, 92). Primary CPP are amorphous accumulations of Ca-P, and their transformation to secondary CPP, composed of crystalline Ca-P, may provide information about the contributor/inhibitor balance (91, 93). T_50_ has been associated not only with more severe CAC and progression in patients with CKD G2–G4, but also with cardiovascular and all-cause mortality in individuals with ND-CKD, hemodialysis and transplant patients (94–97). T_50_ could also reflect factors that promote intimal calcification (98) since secondary CPP stimulates inflammation and apoptosis of macrophages, which may promote ectopic calcification (99, 100). Actually, early stages of CKD were associated with local up-regulation of proinflammatory and pro-osteogenic molecules in the vascular wall and calcification of the aortic media layer (101). Moreover, Ca × P product correlated well with markers of inflammation, but not with calcification itself (101). In fact, Takx et al. (102) recently conducted a retrospective study including CKD-G3 patients and matched controls who underwent fluorodeoxyglucose-PET or CT imaging and concluded that moderate CKD is associated with arterial inflammation, independently of subclinical atherosclerosis. On the other hand, Joshi et al. (103) demonstrated the presence of VC at baseline to be associated with progressive calcification and suggested that VC can be the *inducer* of inflammation and not only its consequence. Of note, hyperphosphatemia, among other mineral metabolism parameters, has also been independently associated with inflammation and VC in CKD (104, 105). Thus, T_50_ may not directly reflect the initial pathophysiological pathway of VC but could theoretically help in clinical decisions on CKD-MBD-related treatments and/or identifying high-risk patients (97). Similarly, the so-called fetuin-A (VC inhibitor) reduction ratio was also evaluated as a potential parameter to measure extraosseous calcification stress (97, 106, 107).

## Treatment Implications

Although VC is a surrogate marker and is not yet considered a treatment target in CKD (108), the presence of VC may have important therapeutic implications (34, 35, 44), unless all patients are treated with the mindset of reducing the incidence or progression of VC (i.e., using all minimization strategies such as reducing exogenous Ca and P).

### Phosphate Binders

Several studies have shown that non-Ca-based P binders, mainly sevelamer in dialysis patients, attenuate the progression of valvular and VC as compared to Ca-based P binders (109–112). Non-Ca-based P binders have also been associated with improved survival (109, 113–115), although certainty of evidence is low (116). Russo et al. (117) randomized 90 non-diabetic ND-CKD G3-G5 patients without a history of CVD to three groups: low P-diet only, Ca-based P binders, or sevelamer. They monitored patients for an average of 2 years and found that CAC significantly increased in patients on the low-P diet alone, to a lesser extent in Ca-carbonate-treated patients, and not at all in sevelamer-treated patients (117). Moreover, Di Iorio et al. found in 212 patients with CKD G3-G4 that sevelamer provided benefits in all-cause mortality and in the composite end point of death or dialysis inception (118). Interestingly, considering only patients with CAC>0, a significant regression of CAC was observed in more patients treated with sevelamer than Ca-carbonate (24 vs. 2). Over 24 months, the final cumulative percentage of *de novo* onset of CAC was 12.8 and 81.8%, respectively (118). Consequently, recent guidelines (35, 44) increased the degree of evidence from 2C to 2B, suggesting that in adult patients with CKD G3a–G5D the dose of Ca-based P binders should be restricted.

In these guidelines, lowering elevated P levels only toward the normal range is also suggested, mainly because of an absence of data supporting the benefit of efforts to maintain P in the normal range (e.g., in G3a–G4 patients) and also some safety concerns (35, 119). Therefore, guidelines now suggest that treatment should only aim at overt hyperphosphatemia and emphasize that early “preventive” P-lowering treatment is currently not supported by data (35). Nevertheless, recent studies have analyzed the potential of “preventive” treatment of early P loading in CKD patients (120, 121). Bouma-de Krijger et al. (120) found that sevelamer carbonate for 8 weeks did not induce a significant reduction in PWV and that serum FGF23 did not decrease despite a decline in 24-h urine P excretion. These findings challenged to some extent the concept that “preventive” P binder therapy is a useful approach, at least over this short period. Interestingly, in a subgroup of patients with absent or limited abdominal VC, treatment did result in a statistically significant reduction in adjusted PWV, suggesting that PWV is amenable to improvement in this subset (120, 122). On the other hand, Toussaint et al. (121) recently reported that lanthanum carbonate did not affect arterial stiffness or VC in CKD G3b-4 patients; however, an absence of a significant decline in 24-h urine P excretion seems difficult to explain. Therefore, interpretation of the scarce and heterogeneous observations described in early CKD remains difficult, and the possibility of beneficial effects of a “preventive” treatment may not yet be completely disregarded (122). Finally, significant beneficial effects of a fixed dose of ferric citrate in advanced ND-CKD patients merit further study (123), since it was not a placebo-controlled randomized clinical trial (RCT), about 1/3 of patients in the “standard of care” arm took Ca-based P binders, and the outcome was a composite end-point.

### Treatment for Hyperparathyroidism

Recent guidelines also state that in patients with ND-CKD G3a–G5, the optimal PTH level is not known (35, 44). However, an association between improvement of PTH levels with all-cause mortality has not only been described in dialysis but also in ND-CKD patients (124–126). Guidelines also suggest that patients with intact PTH levels progressively rising or persistently above the assay's upper normal limit should be evaluated for modifiable factors, including hyperphosphatemia, hypocalcemia, high P intake, and VD deficiency (evidence 2C) (35). In fact, low calcidiol levels represent a novel cardiovascular risk marker and have been directly associated with the presence and progression of VC and decreased survival (127, 128). VD may have atheroprotective effects; however, clinical studies do not show improved survival with VD administration (129).

Supplementation with native (nutritional) VD has been shown to have beneficial effects on several biological parameters, even in dialysis patients (130, 131), but a significant RCT with positive hard-outcomes is lacking. On the other hand, in adult patients with ND-CKD G3a–G5, recent guidelines (35) suggest that calcitriol and VD analogs should *not* now be routinely used (evidence 2C), now considering reasonable to reserve them for patients with CKD G4–G5 with severe and progressive hyperparathyroidism (35). Recent RCTs of VD analogs failed to demonstrate improvements in clinically relevant outcomes and an increased risk of hypercalcemia (132, 133). However, very high doses of paricalcitol and/or Ca-based P binders were frequently used in those studies (132, 133). All guidelines agree that modest increases in PTH may represent an appropriate adaptive response in CKD and “progressively rising” PTH levels (trends) rather than PTH “above the upper normal limit” should be considered for treatment (35, 44). Nevertheless, other guidelines do not consider it acceptable to wait until *severe* hyperparathyroidism is present, and physicians are then advised to avoid hypercalcemia and/or hyperphosphatemia but *not* to aim for complete normalization of PTH (44, 134). In experimental animals, low clinically relevant dosages of calcitriol and paricalcitol seem to protect against CKD-related VC (135). Paricalcitol and calcitriol at equipotent doses also showed different effects on VC (136, 137), and distinct positive pleiotropic effects and survival benefits were described in retrospective studies in dialysis patients (138, 139); however, they have not been demonstrated in RCTs.

On the other hand, calcimimetics are important contributors to the achievement of biochemical treatment goals (especially Ca and PTH, but also P) in CKD-MBD (140, 141). Calcimimetics have been shown to decrease VC in experimental uremic animals (142–145) and, in the ADVANCE study, Raggi et al. demonstrated that in hemodialysis patients with secondary hyperparathyroidism, cinacalcet plus low-dose VD may attenuate cardiac valve and VC (37). In fact, calcimimetics do not only correct PTH hypersecretion but also modulate the vascular Ca-sensor activity (72). However, cinacalcet has not been approved in ND-CKD patients, although it can be prescribed in patients with *primary* hyperparathyroidism (with or without CKD) as an alternative or bridging therapy to treat hypercalcemia (146–148).

Finally, the fact that a statin paradox exists (statins promoted coronary atheroma calcification but improved clinical outcomes) (149) means that the implication of VC may be reconsidered (150). Nevertheless, many experimental and/or preliminary clinical studies on VC with different compounds are ongoing (151–160). Thus, other factors (directly or indirectly related with CKD-MBD) such as magnesium, vitamin K, inhibitors of intestinal P transporters, bisphosphonates, denosumab, sodium thiosulfate, alkaline phosphatase inhibitors, apabetalone, sotatercept, recombinant BMP-7, as well as metformin, antioxidants, spironolactone, senolytics, zinc, and the recently tested SNF472 (35, 161, 162) have been shown to potentially attenuate VC. However, as it has been mentioned before, prospective RCTs on hard-outcomes are still lacking with any treatment.

## Conclusion

CKD-MBD is a common complication and contributes to the high morbimortality (mainly cardiovascular) in CKD patients. The prevalence of VC (intimal and medial), even in early stages of CKD, is very high and progresses rapidly. Although VC may not be a direct cause of CVD, it may reflect underlying pathophysiologic derangements and does have deleterious hemodynamic consequences linked to a negative prognosis. Several common treatments may increase or attenuate its natural progression; therefore, CKD-MBD treatment requires an integral approach, addressing all its components, although clear evidence that this strategy improves hard outcomes is lacking. Meanwhile, it seems logical to avoid P loading, restrict Ca-based P-binders, avoid high doses of VD, and avoid normalizing PTH levels. The availability of new drugs and future studies including early CKD patients may lead to significant improvements not only in patient stratification but also in slowing the natural progression of VC.

## Author Contributions

JB was invited as an expert in the field as co-coordinator of the Spanish Nephrology guidelines and experience in the field of chronic kidney disease and mineral and bone disorders (pathophysiology and treatment), contributed to the conceptualization and writing and editing of the manuscript. AA contributed to the conceptualization, search and selection of the most important references, and writing and editing of the manuscript, as well as creation of illustrative Figure 1. CA contributed to the search and selection of the most important references and editing of the manuscript. PM, ML, JO, GB, YG-M, and NR contributed to the conceptualization and review and editing of the manuscript. LD'M contributed to the conceptualization and review and editing of the manuscript, as well as the creation of illustrative Figure 2. JG contributed to the conceptualization, review and editing of the manuscript, and he is the first author of the main contribution to the field of vascular calcification in non-dialysis dependent CKD patients.

## Conflict of Interest

JB declares advisory, lecture fees, and/or travel funding from Amgen, Abbvie, Sanofi-Genzyme, Shire, Vifor-Fresenius-Renal Pharma, Rubió, and Sanifit. AA declares lecture fees and/or travel funding from Amgen. PM acknowledges consultation or speaker honoraria from Abbott Nutrition, Amgen, Fresenius-Kabi, Nutricia, Palex, Sanofi-Genzyme, and ViforPharma. ML declares lecture fees from Abbvie and Vifor-Fresenius-Renal Pharma. The remaining authors declare that the research was conducted in the absence of any commercial or financial relationships that could be construed as a potential conflict of interest.
